# Umbilical cord mesenchymal stem cells from gestational diabetes show impaired ability to up-regulate paracellular permeability from sub-endothelial niche

**DOI:** 10.1042/CS20230657

**Published:** 2024-01-12

**Authors:** Samar Salem, Lopa Leach

**Affiliations:** School of Life Sciences, Division of Physiology, Pharmacology and Neuroscience, Faculty of Medicine and Health Sciences, University of Nottingham, Nottingham, U.K.

**Keywords:** Endoglin, HUVEC, Metformin, VE-cadherin, VEGF-A, WJMSC

## Abstract

*In vitro* studies have shown that Wharton’s jelly mesenchymal stem cells (WJ-MSCs) can cross umbilical and uterine endothelial barriers and up-regulate endothelial junctional integrity from sub-endothelial niches. This pericytic behaviour may be lost in pregnancies complicated by gestational diabetes (GDM), where increased vascular permeability and junctional disruption are reported. The aim of the present study was to investigate whether WJ-MSCs isolated from GDM pregnancies displayed any changes in morphology, proliferation, VEGF-A secretion, and their ability to influence paracellular junctional composition and permeability. WJ-MSCs were isolated from human umbilical cords from normal pregnancies (nWJ-MSCs, *n*=13) and those complicated by GDM (gWJ-MSCs), either diet-controlled (d-GDM, *n*=13) or metformin-treated (m-GDM, *n*=9). We recorded that 4-fold more WJ-MSCs migrated from m-GDM, and 2.5-fold from d-GDM cord samples compared with the normal pregnancy. gWJ-MSCs showed a less predominance of spindle-shaped morphology and secreted 3.8-fold more VEGF-A compared with nWJ-MSCs. The number of cells expressing CD105 (Endoglin) was higher in gWJ-MSCs compared with nWJ-MSCs (17%) at P-2. The tracer leakage after 24 h across the HUVEC + gWJ-MSCs bilayer was 22.13% and 11.2% higher in the m-GDM and d-GDM, respectively, HUVEC + nWJ-MSCs. Transfection studies with siRNAs that target Endoglin were performed in n-WJ-MSCs; transfected cells were co-cultured with HUVEC followed by permeability studies and VE-cadherin analyses. Loss of Endoglin also led to increased VEGF-A secretion, increased permeability and affected endothelial stabilization. These results reinforce the pericytic role of nWJ-MSCs to promote vascular repair and the deficient ability of gWJ-MSCs to maintain endothelial barrier integrity.

## Introduction

Mesenchymal stem cells differentiate into and participate in neovasculogenesis of both the umbilical and placental circulation [1,2], displaying a pericytic commitment. They also modify the microenvironment promoting tissue repair processes by cell-to-cell interactions and/or secreted cytokines, including antioxidant, anti-apoptotic and growth factors, such as epithelial growth factors, vascular endothelial growth factor, transforming growth factor α and β, fibroblast growth factor, and insulin-like growth factor type 1 [3–5]. The impact of pregnancy complications like gestational diabetes on the placental vascular function [6,7] and fetal endothelial cells [8] suggests that extraembryonic stem cells such as WJ-MSCs may also be affected. Gestational diabetes mellitus is a common medical complication of pregnancy comprising approximately 90% of diabetic pregnancies [9] and is defined as any grade of glucose intolerance with onset or first diagnosis during pregnancy [10]. GDM is a transient health condition that mostly disappears after pregnancy but its long-term impacts on maternal health and fetal development, predict that WJ-MSCs emerging during early pregnancies may be part of the mechanisms dictating vascular dysfunction. Previous studies revealed the impact of GDM on human umbilical cord-derived stromal cells that showed reduced cell proliferation and mitochondrial activity, lower chondrogenic and osteogenic differentiation potentials, premature aging [11] and increased reactive oxygen species levels [12]. Other studies have revealed the impact of GDM on amniotic mesenchymal stem cells that showed up-regulated expression of genes involved in the inflammatory responses (TNFα, CTSS, CD40, and MCP-1) and the down-regulated IL-33 anti-inflammatory cytokine [13], and on the chorionic MSCs-GDM that showed significantly increased adipogenic differentiation ability [14]. Furthermore, in this study, we sub-grouped the GDM group according to the treatment modality, given that Villota et al. reported that metformin has a regulatory effect over occludin expression in GDM pregnancies [6].

WJ-MSCs has been shown to restore uterine and fetal endothelial function, via paracrine secretions and cell-cell contact mechanisms [5]. The proximity between the WJ-MSCs and the HUVECs *in situ* and their developmental origins makes these stem cells more amenable to peri-vascular lineage commitment [15]. The communication between the endothelium and mesenchyme allows perivascular cell formation and stable vessel formation [16]. Ebrahim and Leach (2016) reported that the trans-endothelial migration of WJ-MSCs started by 60 min after a VEGF-A linked phosphorylation of VE-cadherin, loss of latter from junctions and widening of paracellular clefts. The return of full endothelial junctional occupancy by VE-cadherin was a post-migratory event; indeed, there was a higher percentage of adherens junctions (AJs) in these co-cultures compared with control HUVEC monolayers alone. The tightening of paracellular barrier, further evidenced by a reduction in endothelial permeability to hydrophilic tracers, were also concomitant with a decrease in VEGF-A levels in the a forementioned study and that of Pati et al. [15,17]. Thus, our study elaborates on this and includes the confounding effects of gestational diabetes on this phenomenon. The essential role of mesenchymal stem cells in the vasculogenesis of many pathological processes has been confirmed to promote local revascularization via secreting angiogenic growth factors such as VEGF [18]. The disruption of paracellular barriers is a requisite for endothelial cell migration and initiation of angiogenesis [16,19]. The restoration of endothelial barrier integrity by pericytes is important for optimal vascular functioning.

Gestational diabetes has always been considered a mild pregnancy complication, and treatment is linked to glycaemic control (diet or metformin). However, that fetal cells may be epignetically/genetically altered in GDM has not been explored. In our previous study [6], we reported that fetal endothelial cells (HUVEC) in diet-controlled GDM show expression of different occludin isoforms and mRNA expression linked to angiogenesis which was not seen if metformin was used for glycaemic control. The use of umbilical stem cells for therapy should be vigilant to these epigenetic alterations which may be linked to mode of treatment. WJ-MSCs may be a useful indicator of the adverse effects of gestational diabetes on the fetus given they are readily available and are fetal cells. This study, therefore, investigated whether WJ-MSCs from gestational diabetic pregnancies (gWJ-MSCs) are phenotypically different from normal pregnancies, specifically in their ability to migrate from stem cell niches, their morphology, proliferation index, expression of mesenchymal stem markers, ability to transmigrate to pericyte niches by secretion of key vasoactive mediators, and whether their ability to influence endothelial barrier function is dependent on treatment modalities used.

## Methods

### Study population

Term umbilical cords (UCs) were obtained at elective caesarean sections from normal pregnancies and GDM controlled either by diet or metformin (*N*=35, >37 weeks’ gestation) (Table 1) with informed patient consent and full ethical approval (REC: OG010101) Nottingham University Hospitals, NHS Trust UK. This study was carried out in accordance with The Code of Ethics of the World Medical Association (Declaration of Helsinki). Pregnant women with singleton pregnancies were included, and pregnancies complicated with hypertension, pre-eclampsia, pre-gestational diabetes, anaemia, fetal congenital anomalies, and smoking were excluded. Women in both groups were not taking any other medications. Norm glycaemic and gestational diabetes state was confirmed by OGTT between 24 and 28 weeks [20]. Birth weight (BW) was measured for newborns after delivery.

**Table 1:** Clinical characteristics of the study population

Characteristic	Normal (*n*=13)	d-GDM (*n*=13)	m-GDM (*n*=9)
**Maternal age, years**	34.1 ± 6.1	34.9 ± 7.6	34.6 ± 2.8
**Maternal BMI, kg/m^2^**	23.4 ± 3	29.3 ± 6.6	34.7 ± 5.6
**Gestational age at delivery, weeks**	39.5 ± 0.6	38.9 ± 1.1	38.8 ± 0.7
**Placental weight, g**	572.7 ± 59.5	614.4 ± 93	689.9 ± 47
**Baby weight, kg**	3.7 ± 0.6	3.5 ± 0.6	3.9 ± 0.7
**Individual birthweight centile [gender]**			
	61.75^th^[F]	74.24^th^[M]	92.64^th^[F]
	46.68^th^[M]	63.47^th^[F]	97.15^th^[M]
	70.73^rd^[M]	89.23^rd^[M]	76.98^th^[M]
	96.49^th^[F]	95.15^th^[F]	64.85^th^[F]
	52.25^th^[F]	99.87^th^[M]	89.65^th^[F]
	93.55^th^[F]	86.68^th^[F]	96.31^st^[F]
	47.34^th^[F]	78.17^th^[M]	100.00^th^[M]
	98.05^th^[M]	98.52^nd^[F]	70.14^th^[F]
	76.75^th^[F]	87.41^st^[F]	91.36^th^[F]
	55.94^th^[M]	81.31^st^[M]	
	76.44^th^[F]	88.33^rd^[F]	
	47.35^th^[F]	92.37[M]	
	98.06^th^[M]	47.23^rd^ [F]	

Data are presented as mean ± SD. [F] neonatal sex female, and [M] neonatal sex male.

### Migration and proliferation of WJ-MSCs

WJ-MSCs were non-enzymatically isolated from UC using the method from [15]. Briefly, UC was divided into small pieces (5cm), each piece was cut longitudinally away from the blood vessels to expose WJ, small incisions were made in the inner surface, the cord pieces were washed with sterile saline/antibiotic solution. Each piece was placed in a separate 10 cm Petri dish with the WJ facing downwards, left to attach to the plate surface, and 15 ml DMEM/low glucose (Sigma Aldrich, U.K., catalog# D6046), 100 IU/ml penicillin, 100 µg/ml streptomycin, 0.25 µg/ml amphotericin B (Fisher Scientific, U.K., catalog# 11536481) and 15% Fetal Bovine Serum (Sigma Aldrich, U.K., catalog# F9665) were added. The number of migrated cells from three UC pieces from normal pregnancy and GDM groups was recorded (fixed dimensions) by taking images of all the cells after specimen removal on day 7 and counted by ImageJ software. To assess cells’ proliferation, WJ-MSCs at 90% confluence, were harvested from T-75 after trypsinisation. The cells were centrifuged at 1000 rpm, resuspended, stained with trypan blue, and counted by TC20 Automated Cell Counter. Doubling time was calculated using the following equation [21]:

$$\text{Doubling Time}\;=\;\frac{\text{Duration}\times \log\;(2)}{\log\;(\text{Final Concentration})\;–\;\log\;(\text{Initial Concentration})}$$

### Characterization of MSCs using flow cytometry

WJ-MSCs were detached when 90% confluent, washed in PBS+ 1% FBS buffer and re-suspended at a concentration of 1 × 10^7^ cells/ml. Cells were stained with FITC Mouse Anti-Human CD90, PE Mouse Anti-Human CD44, PerCP-Cy™ 5.5 Mouse Anti-Human CD105, APC Mouse Anti-Human CD73, PE Mouse Anti-Human CD14, CD 19 and HLA-DR using the Human MSC analysis kit (BD Biosciences, catalog# 562245) (1:20) as prescribed by the manufacturer’s instructions. Isotope control for each fluorophore and unstained cells (negative control) were also used. Cells were analysed on the flow cytometer BD FACS CANTO II. Results were analyzed using Kaluza software and presented as histograms.

### ELISA assay for secreted VEGF-A

The culture supernatant was collected from confluent WJ-MSCs on T-75 flask. The number of cells was counted. The supernatant was centrifuged at 1900 rpm for 10 min at 4°C. ELISA assay was done using Human VEGF Quantikine ELISA Kit (R&D Systems, U.S.A., catalog# DVE00) as prescribed by the manufacturer’s instructions and calculated per 1×10^6^ cells. The samples were run in duplicates, and a standard curve was created to interpolate the sample concentration using Prism software.

### Transmigration studies

HUVECs were isolated using the method from [22]. Briefly, the vein from each end was cannulated by three-way stopcocks and pre-warmed saline was used to remove any residual blood. About 1 mg/ml collagenase type II (Gibco, U.K., catalog# 17101-015)/Medium199 (Gibco, U.K., catalog# 11043-023) + 100 IU/ml penicillin, 100 µg/ml streptomycin (Sigma Aldrich, U.K., catalog# P0781) was injected into the cannulated vein and stopcocks closed. The cord was placed in pre-warmed saline at 37°C for 10 min. Then, the solution was passed back and forth through the vein to ensure that endothelial cells were detached from the vessel wall. HUVECs were collected in the same media with added 20% heat inactivated FBS, centrifuged at 1000 rpm for 5 min at RT, then re-suspended in the same media with added 75 µg/ml Endothelial cell growth supplement (Sigma Aldrich, U.K., catalog# E2759) and 50 µg/ml Heparin sodium salt (Sigma Aldrich, U.K., catalog# H3149) on gelatin-coated culture plates (Sigma Aldrich, U.K., catalog# G9391). HUVECs were only used up to the third passage in all experiments.

For the co-culture experiments, 100,000 of HUVEC were seeded on 1% gelatin-coated coverslips and incubated in endothelial growth media (EGM) till they reached 70% confluence, then the media was changed to mixed media containing: 50% EGM and 50% stem cell media. When the HUVEC reached full confluence, 20,000 PKH-26 red dye-labelled nWJ-MSCs or gWJ-MSCs (Sigma Aldrich, U.K., catalog# PKH26GL) were seeded on top of the monolayer.

### Immunocytochemistry of Junctional VE-Cadherin in the bilayer

HUVEC and WJ-MSC bilayers were fixed with 4% PFA at 2 and 24 h. Cells were permeabilized with 0.15% triton X-100, blocked with 5% goat serum for 30 min at RT, then incubated overnight at 4°C with mouse anti-human VE-cadherin (10 µg/ml) (BD Biosciences, U.K. catalog# 5556661). After washes, cells were incubated for 2 h in the dark with goat anti-mouse IgG-FITC (Sigma Aldrich, U.K., catalog# F0257) (1:100). Unbiased ten images per coverslip were obtained with Nikon fluorescence microscope with appropriate filters to allow visualization of PKH26 and FITC (duration of co-culture and sample ID were blinded). Images were used to count paracellular junctions and characterised according to VE-cadherin staining pattern at cell-cell borders: continuous or disrupted. A grid was used by ImageJ software so that all the junctional regions had the same chance of being counted, and junctions from every other square which did not cross the ‘forbidden line’ were analysed [23,24].

### Effect of nWJ-MSCs and gW-MSCs on the permeability of HUVECs monolayer

About 5 × 10^4^ HUVECs from normal donors were seeded on 1% gelatin-coated 1.12 cm^2^ culture area transwell inserts (0.4 µm pore) (Corning U.S.A., catalog# 3401) and upon confluence, 10,000 WJ-MSC (Normal and GDM groups) were added on top of the HUVEC monolayer in phenol-free mixed media. FITC-Albumin (1 mg/ml) (66 kDa albumin; Sigma-Aldrich, U.K., catalog# A9771) was added to the apical compartment, and albumin without FITC (1 mg/ml) was added to the basal compartment to maintain equal osmotic pressure. To maintain equal hydrostatic pressure 500 and 1500 μl media were added to the apical and basal compartments, respectively. Fifty microlitres samples were collected from the basal chamber at 0, 4 and 24 h intervals, with the same volume of fresh medium, replenished after each sample collection. Samples were assessed by a fluorescence plate reader (SpectraMax M2e) using the SpectraMax Pro7 application. The FITC–albumin concentration in each sample was calculated via linear regression of a serial dilution series of the tracer. The data are expressed as the amount of leaked protein to the basal compartment. Three replicates were performed per sample.

### Transfection of WJ-MSCs by SiRNA Endoglin (CD105)

The cells were transfected with Silencer Select Pre-designed siRNAs; (s4677 siRNA-1 sequence 5′-UGACCUGUCUGGUUGCACAtt-3′) and (s4679 siRNA-2 sequence 5′-CAAGUAUGAUCAGCAAUGAtt-3′) (Ambion, Life Technology catalog# 4392420) or Silencer® Select Negative Control #1 siRNA (Ambion, Life Technology catalog# 4390843) and Silencer® Select Negative Control #2 siRNA (Ambion, Life Technology catalog# 4390846) in the presence of Lipofectamine™ RNAiMAX Transfection Reagent (Invitrogen, catalog# 13778030) diluted in Opti-MEM™ I Reduced Serum Medium (Gibco, U.K. catalog# 31985062), according to the manufacturer’s instructions. The silencer select negative controls were siRNAs with sequences that do not target any gene product. The cells were then incubated at 37°C for 24 h before replacing the transfection media with complete media without antibiotics. The supernatant was collected from the culture well after 48 h (2 ml) for ELISA quantification of VEGF. To confirm the ENG silencing, 72 h post-transfection the cells were harvested, washed, resuspended in 100 µl PBS+ 1% FBS buffer, and stained with 5 µl CD105 (Endoglin) Monoclonal Antibody (SN6), PE (eBioscience, catalog #12-1057-41) for 30 min at RT in the dark. The stained cells were washed twice before being re-suspended in 300 µl buffer to be ready for analysing by CANTO II cytometer. Specific silencing was confirmed in three independent experiments.

### Effect of ENG silencing on the permeability of HUVEC monolayers

WJ-MSCs (ENG^+/+^ and ENG^−/−^) were placed on top of confluent HUVEC monolayers grown on transwell inserts. After 24 h, when the stem cells have transmigrated, FITC-albumin (1 mg/ml) was added to the apical chamber and the leaked tracer was quantified at the basal compartment (Figure 7A). The data are expressed as the amount of leaked protein to the basal compartment in the two co-culture groups (HUVEC+ ENG^−/−^ WJ-MSCs and HUVEC+ ENG^+/+^WJ-MSCs). For immunostaining, PKH67 green dye-labelled WJ-MSCs (ENG^+/+^ and ENG^−/−^) (Sigma Aldrich, U.K., catalog#PKH67GL) were placed on top of confluent HUVEC monolayers, and the co-culture bilayers were fixed and stained for VE-cadherin. After washes, cells were incubated for 2 h in the dark with goat anti-mouse IgG-TRITC (Sigma Aldrich, U.K., catalog# T5393) (1:100) and the continuous junctions were counted.

### Statistical analysis

Data were tested for normality of the distribution by the D’Agostino–Pearson test with a confidence interval of 95%. Study population, phenotypic characterization, Endoglin expression and tracer leakage were normally distributed. Statistical comparisons between two groups were performed by independent samples *t*-test, and among the three groups by one-way ANOVA. Results of birth weight in all groups, cells’ migration, and VEGF-A secretion in gWJ-MSCs were not normally distributed, performed by Mann–Whitney *U-*test. Pearson’s or Spearman’s rank correlation coefficients were used depending on the normality distribution of the data. The results of flow cytometry assays, transmigration studies, permeability assays, and transfection studies shown were representative of three independent experiments.

## Results

### Comparison of maternal and neonatal characteristics

**Figure 1:**
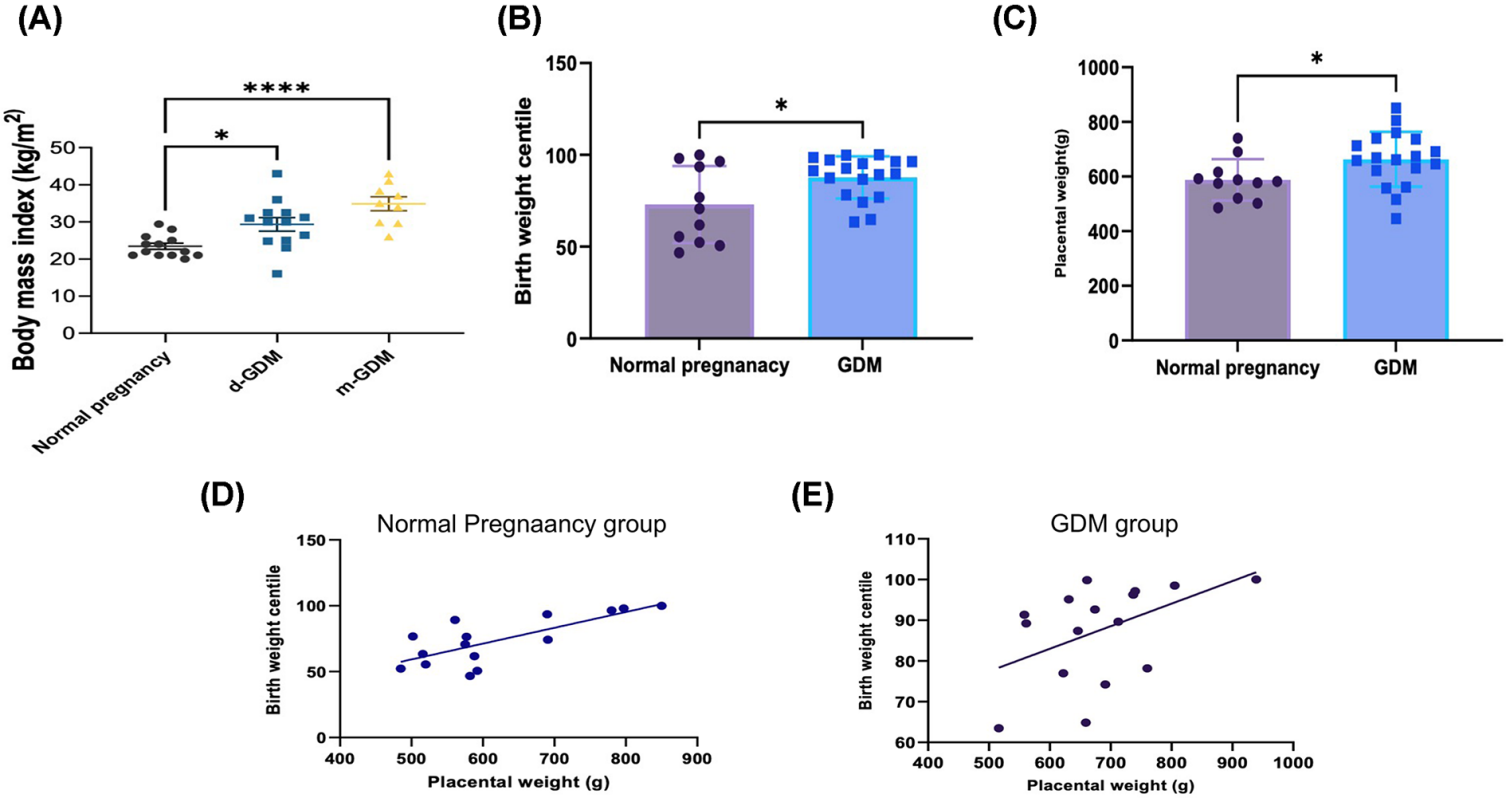
Clinical characteristics of the study population (**A**) BMI of mothers in the GDM group was significantly higher than normal pregnancy. Data represented as Mean (SD). One-way ANOVA was used to compare the groups, ***p < 0.001 by post hoc unpaired t test (**B** and **C**) Graphs displaying increased birth weight centile and placental weight in the GDM group compared with the normal pregnancy group. Data represented as Mean (SD) *p < 0.05 by unpaired t test (**D,E**) Placental weight was positively correlated to the birth weight centile in the normal (Pearson, *r* = 0.8)(***p < 0.001) and GDM group (Spearman, *r* = 0.6) (*p < 0.05) .

Maternal BMI in the study population showed a significant increase in d-GDM (29.3 kg/m^2^ ± 6.6) (*P*-value < 0.005), and m-GDM groups (34.9 kg/m^2^ ± 5.6) (*P*-value<0.0001) compared with BMI in the normal pregnancy group (23.4 kg/m^2^ ± 3) (Figure 1A). The HbA1c from all women in the GDM group showed normal values below 5.7%.

Birth weight displayed no statistical difference between the groups, but birth weight centile corrected for the gestational age was higher in the GDM group (87.67± 11.5) compared with the normal pregnancy group (72.9 ± 16.3) (*P*-value < 0.05) (Figure 1B). Placental weight was higher in the GDM group (642.7 g ± 85.9) compared with the normal pregnancy group (572 g ± 59.5) (*P*-value < 0.05) (Figure 1C). Placental weight was positively correlated to the birth weight centile in both normal pregnancy (Pearson, *r* = 0.8) (*P*-value < 0.001), and GDM group (*P*-value < 0.05) (Spearman,* r* = 0.6) (Figure 1D,E) and did not correlate with any of the maternal variables considered (maternal and gestational ages).

### gWJ-MSCs display altered behaviour: migration, morphology and proliferation studies

**Figure 2:**
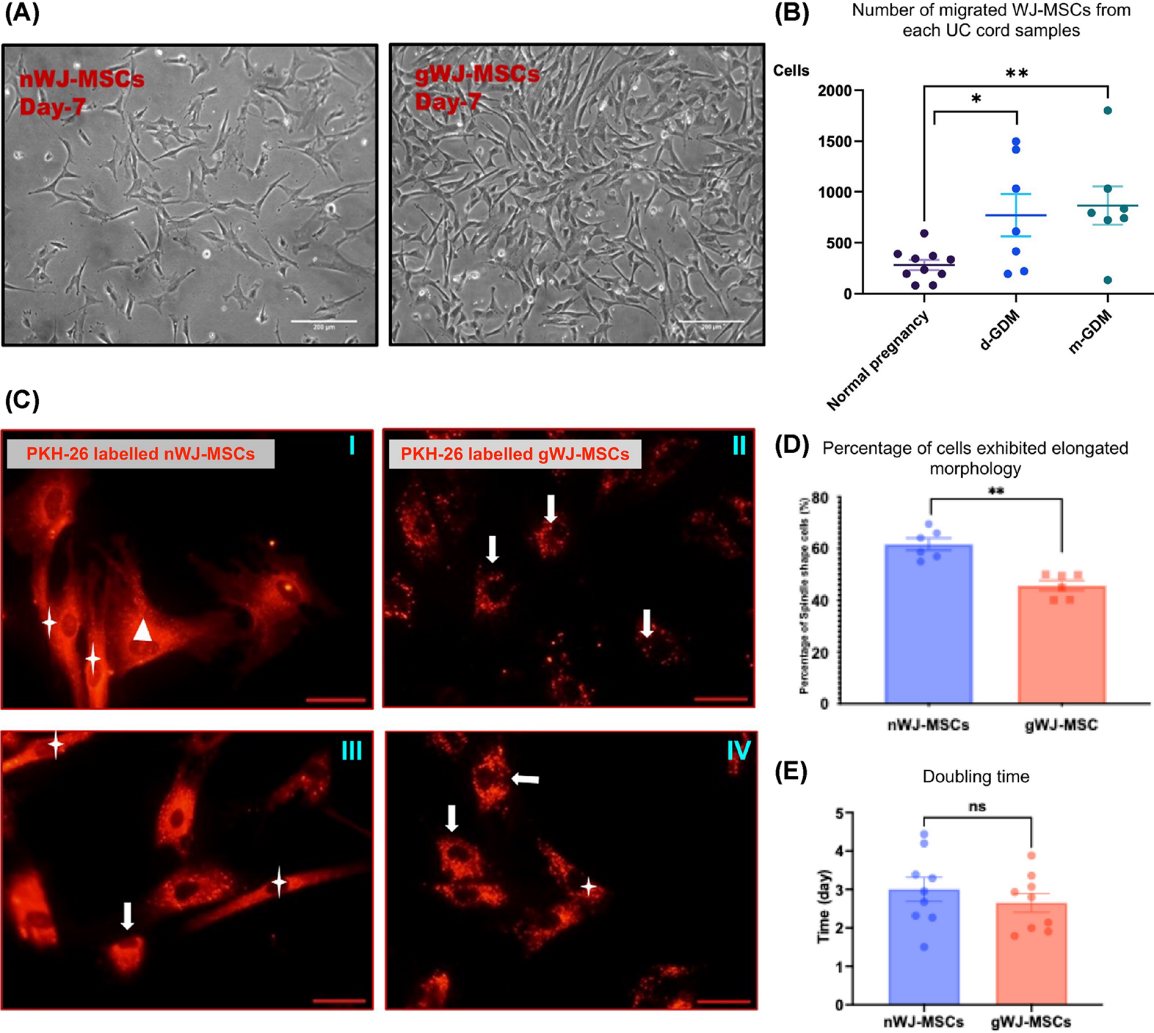
WJ-MSCs migration from cord samples, morphological assessment, and proliferation (**A**) Representative phase contrast micrographs of primary cultures of nWJ-MSCs and gWJ-MSCs (passage 0) day 7; scale bar: 200 μm. (**B**) Increased number of WJ-MSCs migrated from each UC piece in the GDM samples compared with the normal pregnancy. Data represented as Mean (SD) (one-way ANOVA was used to calculate *P-*value *p < 0.05 and **p < 0.005). (**C**) Representative micrographs displaying the mixed morphology of PKH-26 red labelled MSCs (passage 2) with their elongated (star), rounded (arrow), and triangular (triangle) morphology of nWJ-MSCs (images I and II) and gWJ-MSCs (images III and IV) (scale bar = 40 µm). (**D**) Decreased percentage of WJ-MSCs exhibiting the elongated spindle-shaped cells after 24 h. culture in the GDM group compared with the normal pregnancy group (**p < 0.005 by unpaired *t* test ). (**E**) Graph displaying no significant difference between the nWJ-MSCs and gWJ-MSCs in doubling time calculation; *P-*value = 0.4. Data represented as Mean (SD).

The migratory properties recorded at passage (0), by counting the number of MSCs migrating from each UC piece, showed differences between nWJ-MSCs (282.2 ± 49.48) and gWJ-MSCs from the d-GDM group (769.7 ± 207) and m-GDM group (864.9 ± 187.6). The diet-controlled GDM and metformin-treated GDM were 2.5- and 4-fold higher compared with the control (normal pregnancy); however, there was no significant statistical difference between the two treatment modes (*P*-value = 0.1456) (Figure 2A,B). WJ-MSCs isolated from normal and GDM groups exhibited similar elongated fibroblast, rounded and triangular morphologies. They maintained all three morphological types during subsequent passages. However, in sub-cultures, gWJ-MSCs showed a significantly lower percentage of elongated spindle-shaped cells after 24 h (43.8%) compared with nWJ-MSCs (64.1%, *P*<0.005) (*n*=5) (Figure 2C,D). WJ-MSCs from GDM group showed no significant difference in the doubling time (3.3 ± 0.4) compared with nWJ-MSCs (Figure 2E).

### Phenotypic characterization of MSC markers: gWJ-MSCs show fidelity of expression bar expression of CD105

**Figure 3:**
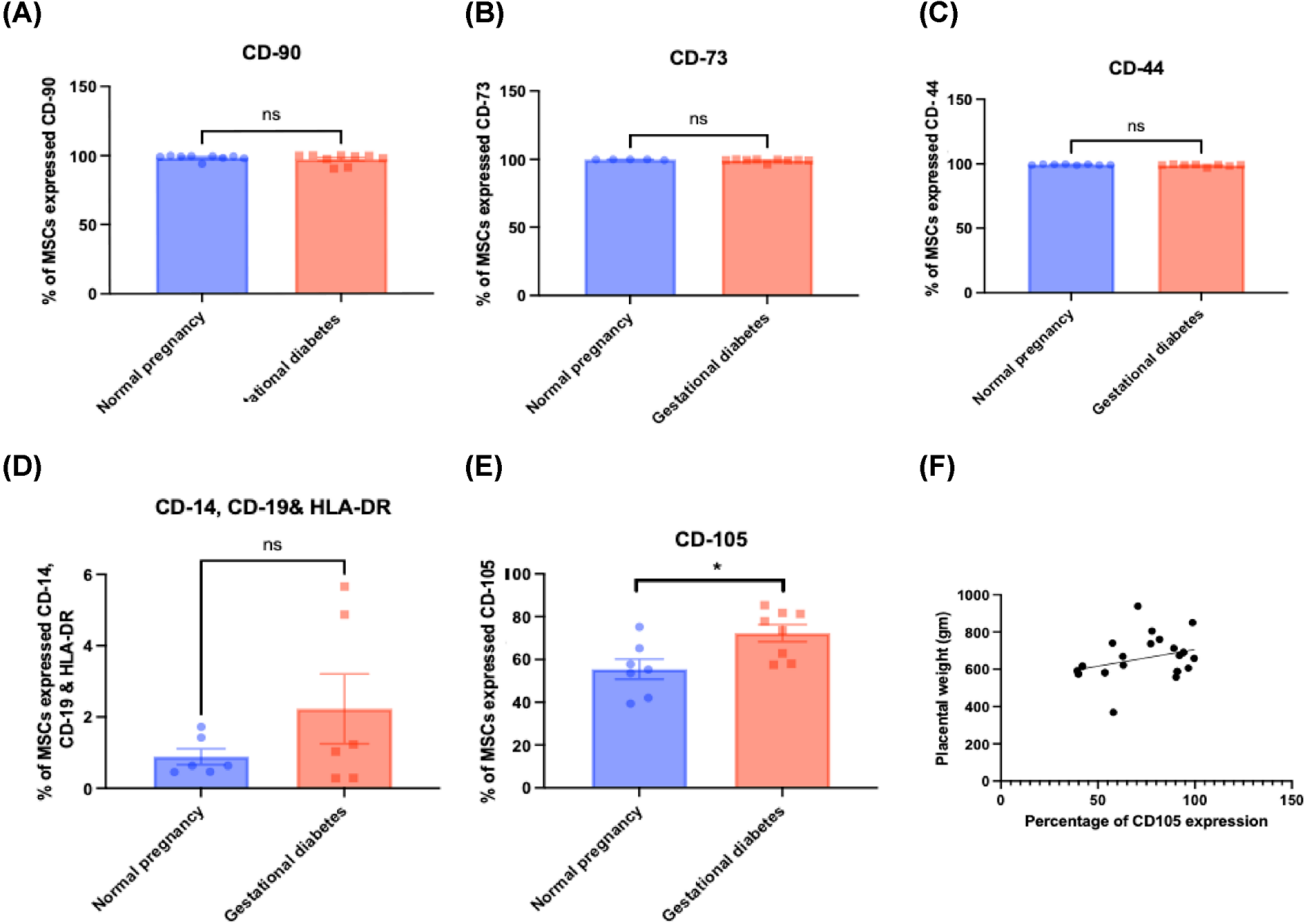
Phenotypic characterization of WJ-MSCs from normal and GDM pregnancies (**A–D**) Graphs displaying no significant difference between Normal and GDM groups in CD90, CD-44, CD73, CD14, CD19, and HLA-DR expression (passage-2). (**E,F**) graphs displaying the increased percentage of Endoglin expression in gWJ-MSCs compared with nWJ-MSCs (passage-2)(*p < 0.05) and the correlation between increased placental weight and increased CD105 expression (Pearson *r* = 0.4) (*p < 0.05), respectively. Data represented as Mean (SD). Unpaired *t*-test was used to calculate *P-*value. Cells were taken from normal pregnancies (*n*=9), gestational diabetes (GDM) (*n*=17) (diet-controlled, *n*=9) and Metformin-treated (*n*=8).

The immunophenotype of nWJ-MSCs and gWJ-MSCs were compared and displayed high levels of expression of typical MSC markers; CD73, CD90, CD105 and low levels of hematopoietic markers; CD14, CD19 and HLA-DR. gWJ-MSCs expressed CD-90 (99.01% ± 1.07) compared with controls (99.4% ± 0.11), CD73 (98.66% ± 1.73) compared with controls (99.71% ± 0.27), and CD44 (98.44% ± 2.059) compared with controls (99.36% ± 0.4622) with no statistical difference between both groups (Figure 3). CD105 expression was higher in gWJ-MSCs (74.12% ± 5.05) compared with nWJ-MSCs (56.56% ± 8.579) at P-2 (*P-*value < 0.05), with no SD between d-GDM and m-GDM (*P*-value = 0.4449). Increased CD-105 was correlated to the increased placental weight in both groups (*P-*value < 0.05) (*r* = 0.4) (Figure 3E,F).

### Differential secretion of VEGF-A from gWJ-MSCs

**Figure 4:**
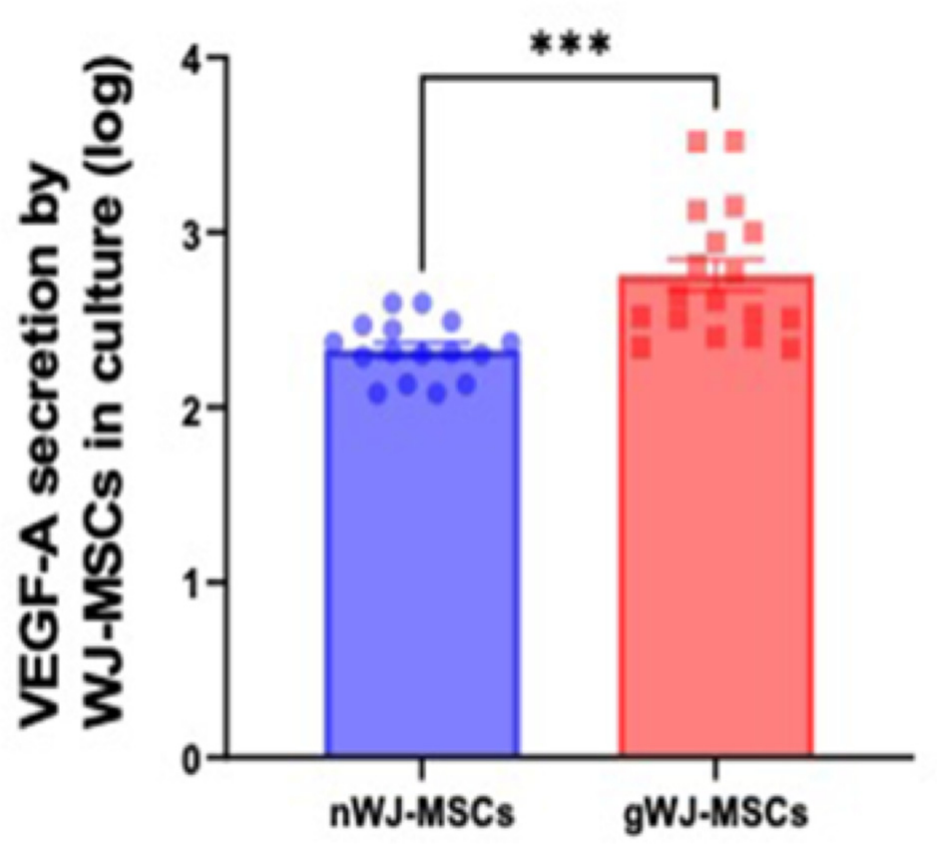
ELISA quantification of VEGF A in the culture supernatant Graph displaying increased VEGF-A concentration in the supernatant of gWJ-MSCs group compared with nWJ-MSCs. Data represented as Mean (SD). Unpaired *t-*test was used to calculate P***≤0.001.

VEGF-A concentration in the culture supernatant was 3.8-fold higher in gWJ-MSCs samples than those from n-WJ-MSCs (*P*≤0.0001) (Figure 4). There was no statistical difference between the m-GDM and d-GDM groups.

### gWJ-MSCs can transmigrate through HUVECs monolayers but show impaired ability to restore junctional VE-cadherin and endothelial integrity (transmigration and tracer permeability studies)

**Figure 5:**
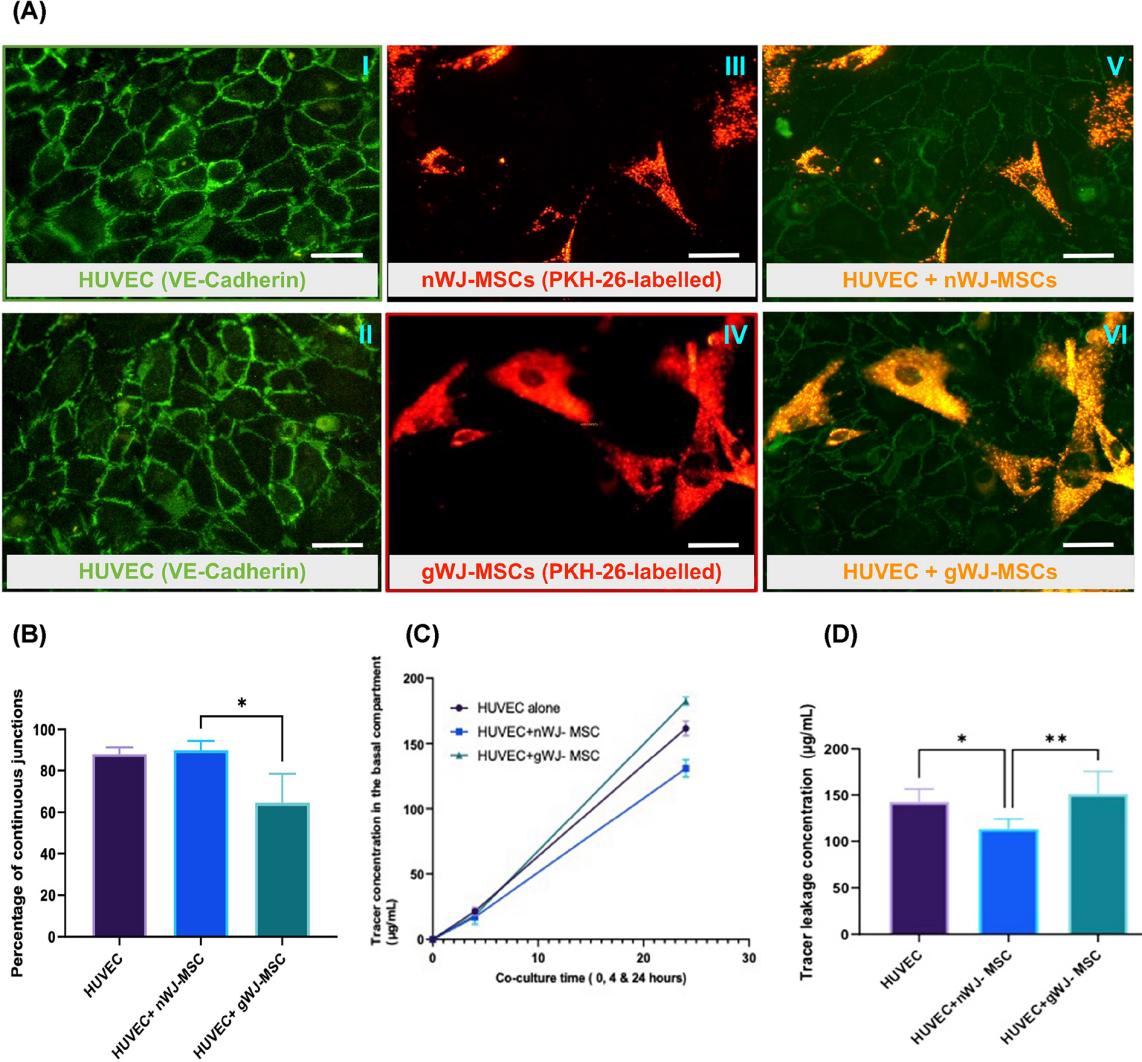
Transmigration of WJ-MSCs across the HUVEC monolayer, VE-cadherin localisation and increased tracer leakage and reduced VE-cadherin junctional occupancy in the co-cultures from the m-GDM group compared with normal and d-GDM groups (**A**) Representative micrographs showing VE-cadherin stained HUVEC (Green) and PKH-26-labelled WJ-MSCs (Yellow) co-cultures. Image (**III**) displaying full VE-cadherin occupancy after 24 hours of co-culture HUVEC + nWJ-MSCs and image (**VI**) displaying reduced junctional VE-cadherin of co-culture HUVEC + gWJ-MSCs compared with the untreated HUVEC monolayer control (**I, II**). Images (**III**, **IV**) showing PKH-26-labelled WJ-MSCs (red) (TRITC filter), scale bar = 40 µm. (**B**) Reduced VE-cadherin junctional expression by 28% after 24 h co-culture of the HUVEC +gWJ-MSCs compared with the HUVEC + nWJ-MSCs,p*<0.05. (**C**) Graph displaying the amount of albumin transferred to the basal chamber across the bilayer cell culture model at 0, 4, and 24 h of co-culture time. No significant difference was found at 0- and 4-h time intervals between the groups (HUVEC, HUVEC + nWJ-MSCs, and HUVEC + gWJ-MSCs), but the difference was observed after 24 h co-culture time. (**D**) Graph displaying nWJ-MSCs decreased tracer leakage while gWJ-MSCs increased it after 24 h co-culture time, p**<0.005. Data represented as Mean (SD). Cells were taken from normal pregnancies (*n*=7) and GDM pregnancies (*n*=10); diet-controlled (*n*=5) and Metformin-treated (*n*=5).

gWJ- MSCs retained the ability to transmigrate across the endothelial monolayer and reside in the sub-endothelial niche. After 24 h in this bilayer arrangement, surface expression of VE-cadherin was found at cell–cell borders of the HUVEC monolayer (Figure 5A). Systematic random sampling of the VE-cadherin staining (continuous vs. disrupted) revealed a 28% reduction in the number of continuous junctions in HUVEC +gWJ-MSCs compared with the HUVEC + nWJ-MSCs (*P*<0.05) (Figure 5B).

The transwell permeability studies showed that tracer leakage to basal compartments were detectable from 4 h in the three groups (HUVEC alone, HUVEC+ nWJ-MSC and HUVEC+ gWJ-MSC), with no significant difference between groups (HUVEC alone : 18.5 μg/ml ± 3.3, HUVEC+ nWJ-MSC :16.7 ± 4.8 μg/ml, and HUVEC+ gWJ-MSC: 18 ± 4.7 μg/ml) (Figure 5D). After 24 h, the tracer leakage values of media from the basal compartment were higher in the gWJ-MSC+HUVEC (151.0 μg/ml ± 8.09) co-cultures compared with nWJ-MSC+HUVEC (113.2 μg/ml ± 4.5) (*P-*value<0.005). Tracer leakage across HUVEC + gWJ-MSC were comparable with HUVEC monolayers alone (142.5 μg/ml ± 4.9) (Figure 5E).

### Endoglin silencing by siRNA transfection of WJ-MSCs

**Figure 6:**
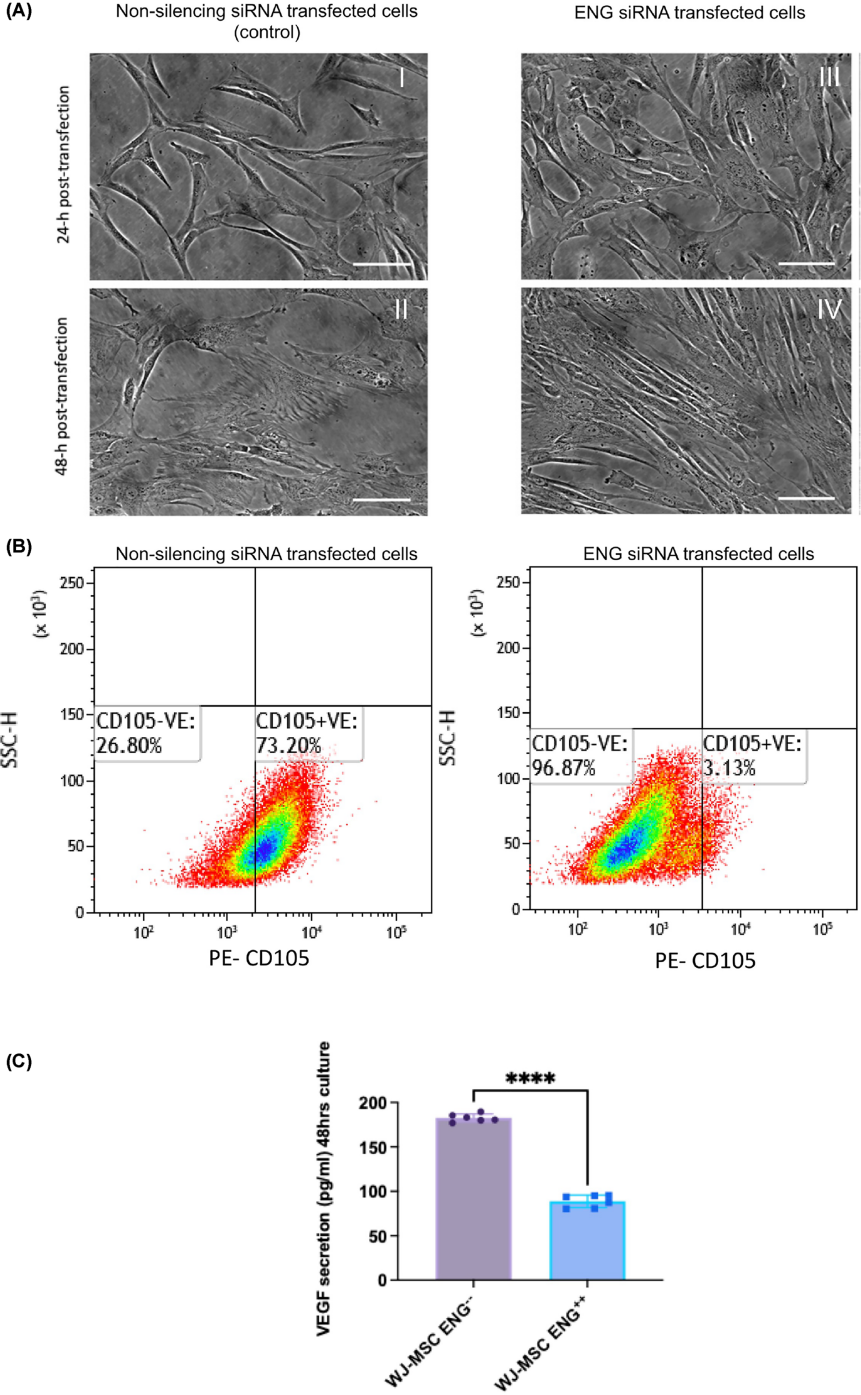
Endoglin siRNAs transfection in WJ-MSCs (P1) (**A**) Representative phase contrast microscopic images of WJ-MSCs displaying no morphology changes after ENG siRNA transfection (images **III** and **IV**) and non-silenced transfected control cells (images **I** and **II**), scale bar = 100 μm. (**B**) Flow cytometry analysis of Endoglin expression after ENG siRNA transfection in WJ-MSCS. Endoglin expression was reduced by 95.6%± 1.9 after ENG silencing, density diagrams displaying the percentage of CD105 expression in WJ-MSC (P-1) after ENG siRNAs transfection and non-silencing siRNAs transfection as a control. (**C**) Graph showing increased VEGF secretion in culture supernatant after Endoglin silencing in the ENG^−/−^WJ-MSCs group compared with the negative siRNA control group. Data represented as Mean (SD) (unpaired *t*-test p****≤0.0001).

ENG expression in WJ-MSCs was reduced by 95.6% ± 1.87 when the cells were incubated with the transfection duplex for 24 h (Figure 6B). Serial transfection for two passages had the same efficiency. ENG-siRNA transfection has no significant effect on the cell viability (87% ± 2.1) compared with the siRNA non-silencing control (90.6% ± 9.5). The Endoglin deficient cells displayed the same morphology compared with the non-silencing-siRNA transfected control cells (Figure 6A). The cells survived the sub-culturing with no significant difference in the calculated doubling time (1.623 ± 0.1614) in ENG^−/−^MSCs compared with its control ENG^+/+^MSCs (1.585 ± 0.4528).

### Endoglin silencing led to increased VEGF-A secretion from ENG^−^/^-^WJ-MSCs

The Endoglin deficient cells ENG^−/−^WJ-MSCs secreted more VEGF-A (184.6 pg/ml ± 4.048) compared with the control group ENG^+/+^WJ-MSCs (87.93 pg/ml ± 8.508) *P-*value≤0.0001, three independent experiments were done to obtain the results (Figure 6C).

### Endoglin silencing led to increased tracer permeability across HUVEC/ENG^−/−^WJMSCs bilayers concomitant with impaired postmigration return of VE-cadherin in HUVEC

**Figure 7:**
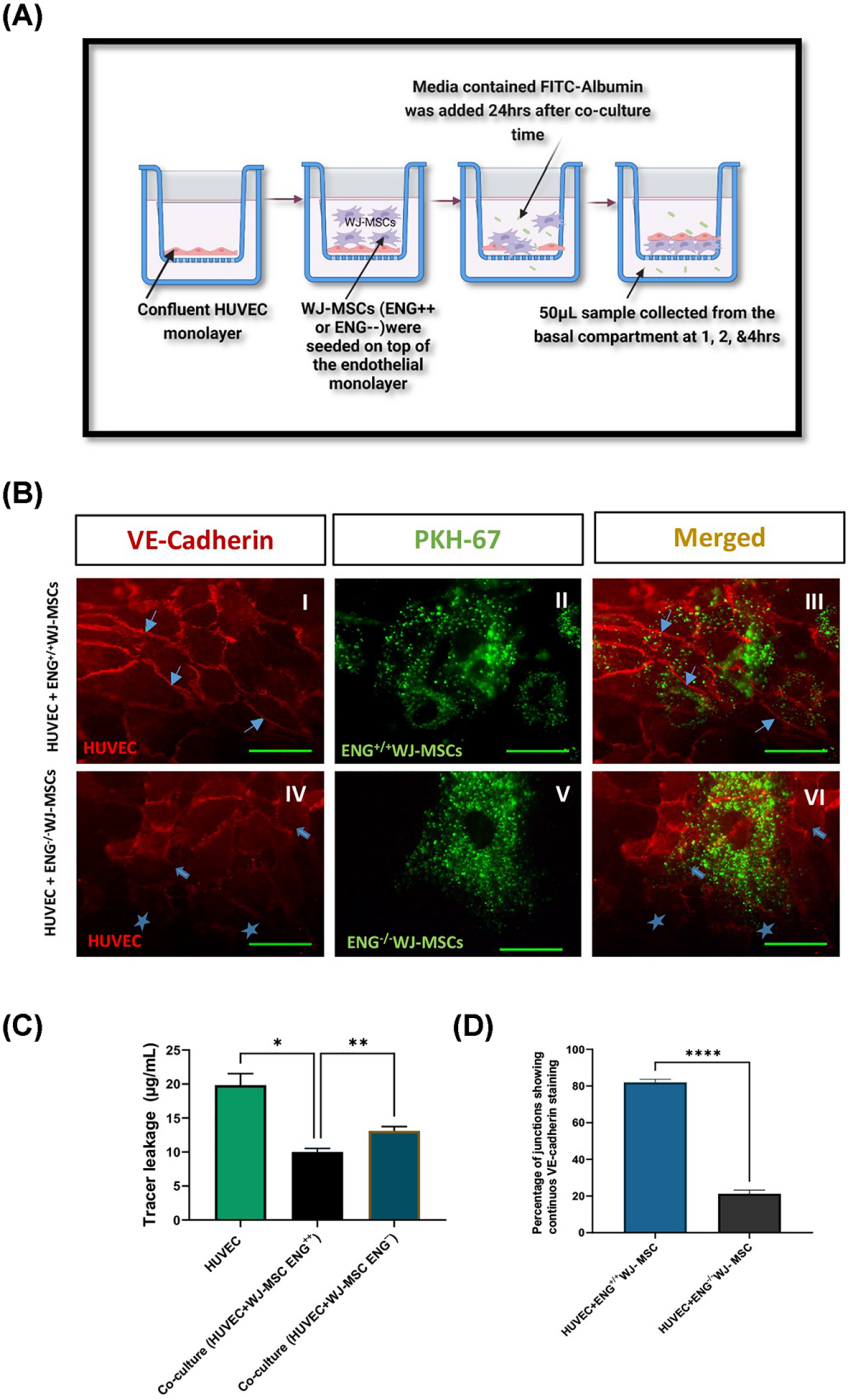
Effect of ENG silencing on the endothelial permeability and VE-cadherin localization in HUVEC+ENG^−/−^ WJ-MSCs (**A**) Diagram illustrating the co-culture permeability assay done after the ENG silencing in WJ-MSCs. (**B**) Representative micrographs displaying VE-Cadherin localisation in co-cultures from HUVEC+ ENG^−/−^MSCs and HUVEC+ ENG^+/+^ MSCs. Images (**I** & **IV**); showing full junctional occupancy (arrows) of VE-cadherin in the HUVECs layer in HUVEC+ ENG^+/+^ WJ-MSCs (**I**) and interrupted (block arrows) and lost junctions (stars) in HUVEC+ ENG^−/−^MSCs (**IV**) (TRITC filter). Images (**II & V**); showing PKH-67 labelled ENG^+/+^ WJ-MSCs (**II**) and ENG^−/−^ WJ-MSCs (**V**) (FITC filter). Images (**III & VI**) are merged images from co-culture HUVEC+ ENG^+/+^ WJ-MSCs (**III**) ENG^−/−^MSCs (**VI**) with the MSCs resting underneath the endothelial cells restoring the VE-cadherin expression. Scale bar = 40 µm. (**C**) Increased FITC-albumin transfer across the co-culture bilayer after ENG silencing. Data represented as Mean (SD). One-way ANOVA was used to compare the groups, p*<05 and p**<0.005. (**D**) Reduced percentage of continuous VE-cadherin junctional counts after 24 h co-culture with silenced WJ-MSCs. Data represented as Mean (SD). Unpaired *t*-test was used to calculate, p****<0.0001.

Permeability assays showed a significant increase in the amount of FITC-albumin that crossed the bilayer over 2 h when HUVEC was co-cultured with ENG^−^ WJ-MSCs (13.11 μg/ml ±1.5) compared with the co-culture HUVEC+ ENG^++^WJ-MSCs (10.01 μg/ml ±1.3), *P*-value≤0.0001. The silencing of ENG resulted in decreasing the ability of WJ-MSCs to maintain the endothelial barrier integrity in the co-cultures (Figure 7C). VE-cadherin showed full junctional occupancy in HUVECs co-cultured with ENG^+/+^ WJ-MSCs. VE-cadherin junctional staining in cell-cell borders were less continuous and more disrupted in HUVEC + ENG^−/−^WJ-MSCs co-cultures (Figure 7B). Counts revealed that the number of junctions displaying continuous VE-cadherin expression was reduced by 75.33% when the HUVECs were co-cultured with the silenced cells (ENG^−/−^WJ-MSCs) compared with the non-silenced HUVEC+ENG^+/+^WJ-MSCs. HUVEC + ENG^−/−^WJ-MSCs displayed a significantly lower percentage of continuous VE-cadherin junctional counts (20.2% ± 8.4) compared with HUVEC+ENG^+/+^WJ-MSCs (81.9% ± 6.7) (*P*<0.0001) (Figure 7D).

## Discussion

In this study, we demonstrated that fetal mesenchymal stem cells are vulnerable to gestational diabetes, regardless of glycaemic control and treatment modality. The phenotypic changes we found are pertinent to vascular function, specifically vascular permeability. G-WJ-MSCs isolated from term umbilical cords appear to have a reduced commitment to a pericytic lineage, which includes a predominance of cell types with rounded rather than elongated morphology, impaired migration of cells from the cords, increased VEGF-A secretion in culture and a continuance of this from sub-endothelial niches. The latter may be behind their inability to restore VE-cadherin localisation post-migration. The continued loss of paracellular integrity of endothelial monolayers resulted in significant egress of FITC-conjugated albumin, a marker of endothelial barrier property to large hydrophilic solutes. G-WJMSCs expressed MSC markers similar to that of n-WJMSCs but displayed higher expression of endoglin. Silencing of endoglin also resulted in loss of function, regarding increased VEGF secretion and restoration of junctional integrity from subcellular locations.

The number of MSCs migrated from umbilical cord pieces from the GDM placenta exceeds those migrated from normal ones, which agrees with Chen et al. who demonstrated enhanced motility of chorionic membrane stem cells from GDM donors and up-regulated expression of AQP1, FLNB, CELSR1, and CD24 genes involved in cell movement (CD 24 & AQP1) [25]. The altered migratory properties seen in gWJ-MSCs suggest that they have similar expression to chorionic fetal stem cells from GDM pregnancy.

The predominant rounded morphology of gWJ-MSCs may be associated with enhanced differentiation potential towards certain lineages. Mcbeath et al. demonstrated that cell shape regulates the commitment of MSCs to adipocyte or osteoblast fate by modulating endogenous RhoA activity [26]. Adipocytes are round and fat-laden [27], while osteoblasts vary from elongated to cuboidal [28]. Therefore, the more rounded morphology of g WJ-MSCs here suggests they have a higher adipogenic differentiation potential. WJ-MSCs from GDM donors have been shown to display more adipogenesis than the ones from normal gestation [29,30]. This may be behind the association between GDM and increased risk of childhood abdominal adiposity [31].

Our study showed no significant difference in the proliferation between nWJ-MSCs and gWJ-MSCs. A lower proliferative rate and increased population doubling time were shown by Wajid et al 2015, however, this study did not consider the glycaemic control of the GDM donor patients [32]. WJ-MSCs from GDM pregnancy have been shown to display decreased cell growth with earlier cellular senescence [11], again glycaemic control was not reported. In our study all women in the GDM group were monitored and treated with metformin or diet and exercise regime to achieve normal values of HbA1c (below 5.7%).

This study showed that gWJMSCs regardless of treatment (diet or metformin) were positive to CD44, CD90, CD105&CD73 and negative to CD14, CD19, and HLA-DR. Kong et al. showed a similar profile for insulin-treated GDM [33]. In addition, we found that gWJ-MSCs displayed higher ENG expression compared with nWJMSCs. Endoglin is a TGF-β co-receptor that mediates endothelial cell proliferation and migration against flow in response to VEGF [34]. Our study is the first to report that higher Endoglin expression is correlated positively to placental weight, but not to birth weight, maternal weight, or maternal age. This may be linked to the higher VEGF levels, given its pro-angiogenic role. GDM is associated with reduced expression of VEGF R1 but high pro-migratory activation of VEGF R2 reflecting a proangiogenic state [8].

ENG silencing in nWJ-MSCs was followed by a reduced ability to maintain endothelial integrity after transmigration. Loss of Endoglin here also led to less continuous and more interrupted VE-cadherin in the co-cultures. Optimal ENG levels appear essential for normal vascular development and endothelial–mesenchymal communication. Both endothelial cells and WJ-MSCs express Endoglin, on their membrane, with a critical physiological role in the cardiovascular system [35]. The *in vitro* increased secretion of VEGF from ENG-deficient cells provides added evidence that ENG inhibits VEGF signalling. Endoglin sustains the VEGFR2 on the cell surface and prevents its degradation in lysosomes [36] and ENG knockdown promotes the clearance of VEGFR2 from the plasma membrane [37]. Endoglin overexpression has been shown to lead to activation of endothelial cells in tumour tissues which is correlated to metastasis [38]. Paradoxically decreased Endoglin expression can lead to down-regulation of VE-Cadherin, thus both result in an impairment of endothelial stabilization.

nWJ-MSCs were able to enhance the endothelial function by reducing transendothelial permeability whilst gWJ-MSCs showed a deficient ability. Reduced VE-cadherin junctional occupancy in response to the continued VEGF secretion from gWJ-MSCs disrupted the endothelial barrier function. Elevated levels of VEGF-A, loss of junctional VE-cadherin, and increased vascular leakage have been reported in pregnancies complicated by maternal diabetes [16,19,24,39,40]. WJ-MSCs from normal pregnancies do secrete VEGF and TGF- β [5,37,41,42] in levels that maintain endothelial stability. The deficient ability of gWJ-MSCs to enhance the endothelial barrier integrity might be related to the higher Endoglin expression. Ibanez et al. suggested that ENG overexpression in mice keeps endothelial cells in an active phenotype resulting in an impairment of the correct stabilization of the endothelium and the recruitment of mural cells [43]. The integrity of fetoplacental barrier relies on the endothelial paracellular cell junctions and the syncytiotrophoblast to maintain tight regulation of the transport of nutrients and oxygen between the mother and the fetus [44].

In this study, maternal BMI may have influenced the use of Metformin in controlling hyperglycemia. Our findings revealed that WJ-MSCs from both metformin and diet-controlled groups made the endothelial barrier more permeable despite good glycemic control in both study groups. Metformin has been shown to have an enhancing effect on occludin [6] but this appears not to extend to VE-cadherin. It does draw attention to its use during pregnancy as it crosses the placenta with fetal levels similar to maternal levels [45–47].

In conclusion, the data obtained in this study show that the uterine environment during pregnancy has an impact on the biology of WJ-MSCs. Even with a well-controlled glycaemia, gestational diabetes can affect the WJ-MSCs’ properties and influence on endothelial barrier integrity. The observed changes regardless of the treatment modalities suggest gWJ-MSC may not be suitable for therapeutic clinical applications.

## Clinical perspectives

- Background as to why the study was undertaken. Although GDM is a transient health condition that mostly disappears after pregnancy its long-term impacts on fetal development predict that WJ-MSCs, emerging during early pregnancies may be part of the mechanisms dictating vascular dysfunction.Point
- A brief summary of the results. WJ-MSCs from the GDM group displayed enhanced migration, overexpressed endoglin, and increased tracer leakage in the transmigration studies.Point
- The potential significance of the results to human health and disease. We demonstrated that endoglin significantly affects the VEGF signalling and its role in GDM need investigation. The enhanced mobility of gWJ-MSCs may promote their migration toward injured sites; however, their impaired ability to maintain the endothelial integrity may negatively affect any perceived benefit.

## Data Availability

The data that support the findings of this study are available from the corresponding author upon reasonable request.

## Open Access

Open access for this article was enabled by the participation of University of Nottingham in an all-inclusive *Read & Publish* agreement with Portland Press and the Biochemical Society under a transformative agreement with JISC.

## Abbreviations

AJ: adherens junction
ENG: endoglin
GDM: gestational diabetes mellitus
HUVEC: human umbilical vein endothelial cell
UC: umbilical cord
VEGF: vascular endothelial growth factor
WJ-MSC: Wharton’s jelly mesenchymal stem cell
